# Insights into *Klebsiella pneumoniae* carbapenem resistance—a two-year retrospective study from a Romanian tertiary care hospital

**DOI:** 10.3389/fmicb.2025.1728843

**Published:** 2026-01-08

**Authors:** Daniela Tălăpan, Mihai-Octavian Dan, Alexandra Cireşă, Alexandru Rafila

**Affiliations:** 1 Faculty of Medicine, “Carol Davila” University of Medicine and Pharmacy, Bucharest, Romania; 2 Microbiology Laboratory, “Prof. Dr. Matei Balş” National Institute of Infectious Diseases, Bucharest, Romania; 3 “Carol Davila” Clinical Military Emergency Hospital, Bucharest, Romania

**Keywords:** antimicrobial resistance, carbapenem resistance, carbapenemases, *Klebsiella pneumoniae*, last-resort antibiotics, resistance mechanisms

## Abstract

**Introduction:**

Antimicrobial resistance amongst *Klebsiella pneumoniae* isolates imposes a significant clinical and public health burden, requiring a large healthcare expense for the treatment of infections. The last decade has seen an incremental rise in antimicrobial resistance, not only toward carbapenems, but also last-resort antimicrobials, urging the need for collaboration between infectious diseases specialists and microbiology peers toward the development of novel therapeutic methods. This study aims to provide a two-year overview in carbapenem-resistant *Klebsiella pneumoniae* (CRKP) in an infectious diseases tertiary hospital in Romania, focusing on antimicrobial resistance patterns and carbapenemase distribution.

**Methods:**

Clinical samples collected from patients admitted to the “Prof. Dr. Matei Bals” National Institute of Infectious Diseases in Bucharest, Romania, between 1st August 2023 and 31st July 2025 have undergone standard cultivation, bacterial identification and antimicrobial susceptibility testing according to standard laboratory protocol. All carbapenem-non-susceptible *Klebsiella pneumoniae* strains (meropenem minimum inhibitory concentration > 0.125 μg/mL) further underwent extended-spectrum beta-lactamase (ESBL) and carbapenemase production testing via phenotypical methods.

**Results:**

A number of 340 non-duplicate, carbapenem-non-susceptible *Klebsiella pneumoniae* strains have been isolated from various clinical samples. The majority of them (> 90%) exhibited carbapenemase production: *n* = 164, 53.59% double NDM and OXA-48-type carbapenemase producers, followed by NDM (*n* = 74, 24.18%), OXA-48-type (*n* = 44, 14.38%), KPC (*n* = 14, 5.56%) and NDM+KPC (*n* = 7, 2.29%). In addition, 309 strains were deemed ESBL producers. In terms of antimicrobial resistance, more than > 90% of the strains exhibited resistance to fluoroquinolones, third generation cephalosporins, penicillins and monobactams, > 75% resistance to aminoglycosides and novel beta-lactam/beta-lactamase inhibitor combinations and > 70% resistance to colistin and imipenem-relebactam. Overall cefiderocol resistance was 32.05%. Upon classification, 25.00% (*n* = 85) of isolates were multidrug-resistant (MDR), 56.47% (*n* = 192) extensively drug-resistant (XDR) and 17.06% (*n* = 58) were pandrug-resistant (PDR). Moreover, 54.17% (*n* = 104) of XDR isolates were only susceptible to one antimicrobial drug.

**Conclusion:**

Rates of resistance remain concerningly high amongst CRKP isolates, with double carbapenemase producers and XDR/PDR isolates dominating the landscape, implying the foreseeable need for updated means of approaching antimicrobial resistance, as last-resort antimicrobials become less effective through increasing resistance.

## 1 Introduction

*Klebsiella pneumoniae* stands as a leading opportunistic pathogen in invasive infections worldwide, incriminating multiple mechanisms of resistance to antimicrobials, thus limiting clinicians’ means of treatment in already challenging situations, a phenomenon further amplified by the acquisition of resistance genes through plasmid-encoded mechanisms, facilitating the increase in antimicrobial resistance rates (Arato et al., 2021). Currently, healthcare providers face the challenge of strains exhibiting multiple carbapenemase production, coupled with mutations leading to improved microorganism adaptability mechanisms, leading to difficult-to-treat infections in patients already presenting with serious comorbidities, in most cases (Yang et al., 2023; Wang et al., 2024; Ardebili et al., 2023). A 2017 meta-analysis by Xu et al. (2017) revealed a more than doubled pooled mortality rate in patients with carbapenem-resistant *Klebsiella pneumoniae* (CRKP) strains compared to the carbapenem-susceptible group (42.14% versus 21.12%, *p* < 0.001).

From an epidemiological perspective, the latest (2024) version of the European Centre for Disease Control’s (ECDC) Surveillance Atlas of Infectious Diseases, has listed Romania as the 3rd most affected European country in terms of carbapenem-resistant *Klebsiella pneumoniae* strains isolated from invasive infections, with a staggering 50.30% rate of resistance, only surpassed by Greece (60.20%) and Bulgaria (67.60%), a stark contrast to other developed countries, suggesting the need for stronger antimicrobial stewardship and urgent development of treatment alternatives (European Centre for Disease Prevention Control [ECDC], 2025). Currently available last-resort antimicrobials, such as ceftazidime-avibactam (CZA), aztreonam-avibactam (ATM-AVI), cefiderocol (FDC), ceftolozane-tazobactam (CZT) or imipenem-relebactam (IMR) are still considered potent alternatives, however, increased resistance rates to these antimicrobials have recently been demonstrated in hospitals (Dan et al., 2025; Karampatakis et al., 2023).

This study aims to provide an accurate characterization of CRKP distribution in our healthcare setting, an infectious diseases-oriented tertiary-care hospital, highlighting resistance rates to multiple current-use antimicrobial substances, including last-resort antibiotics, carbapenemase and extended-spectrum beta-lactamase (ESBL) production and patterns of resistance pertaining to each carbapenemase type.

## 2 Materials and methods

### 2.1 Study design

This retrospective study was conducted in the Microbiology Laboratory of the “Prof. Dr. Matei Balş” National Institute of Infectious Diseases (NIID) in Bucharest, Romania, a monodisciplinary tertiary hospital, and included clinical samples from patients admitted to our hospital between August 2023–July 2025.

### 2.2 Bacterial culture

All clinical samples (urine, sputum/bronchial aspirates/bronchoalveolar lavage, blood, wound secretions, joint fluids and others) collected from patients admitted to NIID with a suspicion of infection were sent to the laboratory and processed according to the specific Standard Operating Procedures. The specimens were inoculated on Columbia sheep blood agar (COS, ThermoScientific™—Oxoid, Wesel, Germany), chocolate agar with Polivitex (PVX, bioMérieux S.A., Marcy-l’Etoile, France), and lactose agar (CLED, ThermoScientific™—Oxoid, Wesel, Germany), respectively. The inoculated plates were incubated for 18–24 h at 37 °C in an aerobic atmosphere, and then were visually read and interpreted.

### 2.3 Strain identification

All bacterial strains were identified using Matrix-Assisted Laser Desorption Ionisation Time-of-Flight Mass Spectrometry (MALDI-TOF MS) Biotyper (Bruker Daltonik GmbH, Bremen, Germany), which characterize bacterial proteins in whole-cell extracts. Bacterial spectra were analyzed using the Biotyper software version 4.1.

### 2.4 Antimicrobial susceptibility testing

Antimicrobial susceptibility testing (AST) was performed using MICRONAUT-S Gram-negative (customized plate) Romania GN 2 EUCAST (Bruker Daltonik GmbH, Bremen, Germany), followed by interpretation according to the EUCAST (European Committee on Antimicrobial Susceptibility Testing) guidelines versions 13.1, 14.0 and 15.0, respectively. All *Klebsiella pneumoniae* strains with a minimum inhibitory concentration (MIC) to meropenem of > 0.125 μg/mL further underwent extended-spectrum beta lactamase (ESBL) and carbapenemase detection. All carbapenemase-producing strains were tested for susceptibility to cefiderocol using UMIC^®^ (Bruker Daltonik GmbH, Bremen, Germany), as per laboratory protocol.

### 2.5 Enzyme production testing

Phenotypic testing for extended-spectrum beta-lactamase and carbapenemase production has been performed following the EUCAST guideline on detection of resistance mechanisms version 2.0, using lateral flow assays. NG Test/CTX-M Multi (NG Biotech, France) was performed to identify CTX-M production, and Resist-5 O.K.N.V.I. (Coris BioConcept, Belgium) to detect OXA-48-type, KPC, NDM, VIM and IMP carbapenemases.

### 2.6 Quality control

The quality control strains for the AST (Micronaut-S and UMIC^®^ Cefiderocol) were *Escherichia coli* ATCC 25922 and *Pseudomonas aeruginosa* ATCC 27853. The quality control strains were *Klebsiella pneumoniae* ATCC 700603 for the lateral flow assay NG Test/CTX-M Multi and *Klebsiella pneumoniae* ATCC BAA-1705, *Klebsiella pneumoniae* NCTC 13442 and *Klebsiella pneumoniae* NCTC 13443 for Resist-5 O.K.N.V.I.

### 2.7 Data storage and analysis

Preliminary data were extracted from the laboratory’s digital database and further stored in an individual, project-assigned Microsoft Excel database, together with all additional information. Data analysis was performed using Microsoft Excel version 16.66.1 (2022 Microsoft). The Chi square test (two tailed) was used for categorical variables and the results were considered to be statistically significant at *p*-value < 0.05. Pooled resistance rates (PRR) concerning last-resort antimicrobials (cefiderocol, ceftazidime/avibactam, ceftolozane/tazobactam, imipenem/relebactam and colistin) were calculated using the formula PRR = [Σ(resistant isolates for each antimicrobial per carbapenemase)/Σ(isolates exhibiting production of each carbapenemase)*5]*100. Isolates were classified into multidrug-resistant (MDR), extensively-drug resistant (XDR) and pandrug resistant (PDR) categories in accordance with international consensus (Magiorakos et al., 2012).

## 3 Results

During the 24 months collection period, 340 non-duplicate *Klebsiella pneumoniae* strains with diminished susceptibility to carbapenems were isolated, harvested from various clinical specimens collected from patients admitted to our healthcare units.

### 3.1 Cohort demographics

All 340 non-duplicate strains have been isolated from individual patients, comprising of 34.70% (*n* = 118) female patients and 65.30% male (*n* = 222). Only two pediatric cases have been recorded, the median age of the cohort being 65.10 years, with a standard deviation of 16.28 years.

### 3.2 Infection sites

Strains were isolated from multiple infection types, with urinary tract infections (UTIs) being the most commonly encountered (69.41%), followed by skin and soft tissue infections (14.12%) and respiratory tract infections (9.12%). The full distribution of infection sites may be found in Table 1.

**TABLE 1:** Infection sites of the 340 isolates strains.

Infection site	Number (*n*)	Percentage (%)
Urinary tract	236	69.41%
Wound secretions	48	14.12%
Respiratory tract (including sputum, bronchial aspirate, bronchoalveolar lavage)	31	9.12%
Bloodstream	20	5.88%
Others (cerebrospinal fluid, joint fluid, sonication probes)	5	1.47%

### 3.3 Carbapenemase production

**FIGURE 1:**
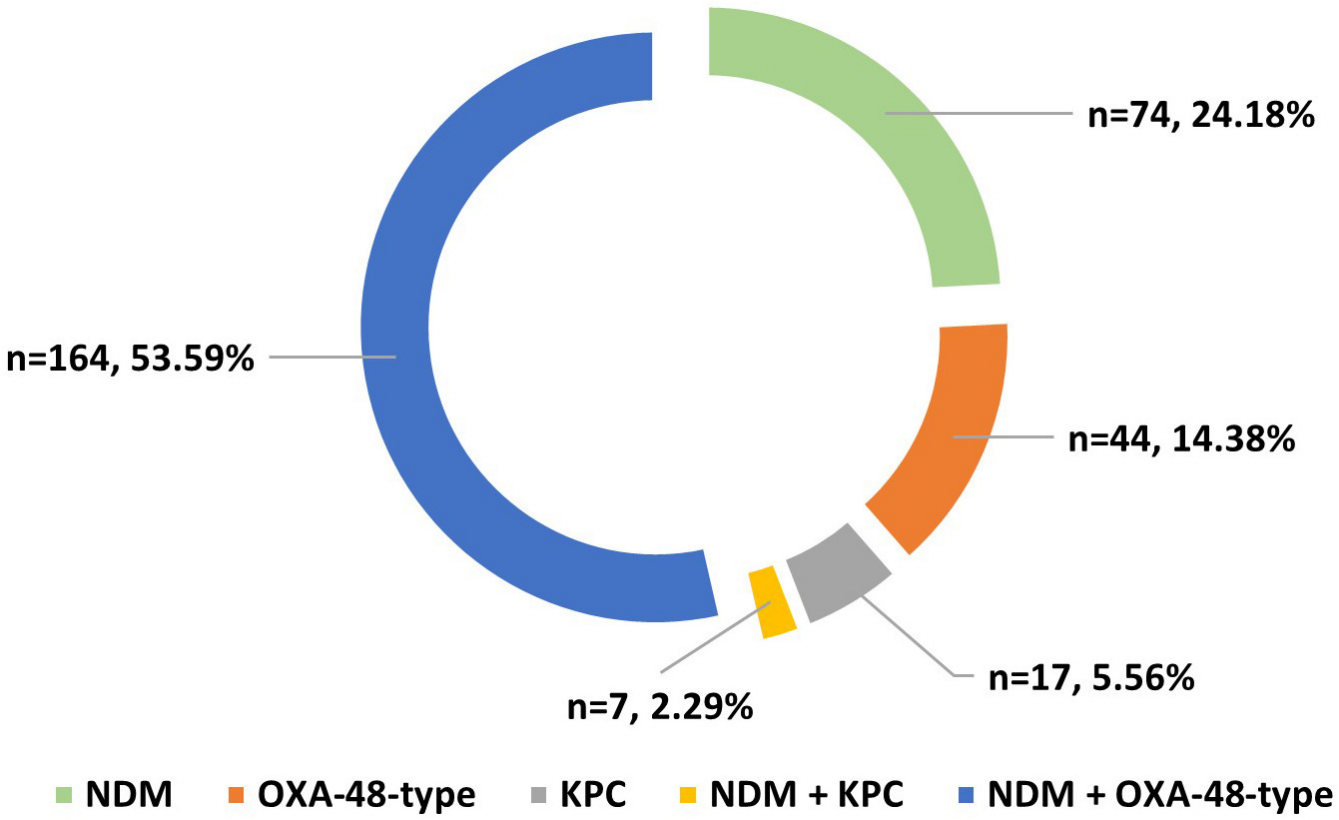
Distribution of carbapenemase types produced by isolates. NDM, New-Delhi metallo-beta-lactamase; OXA-48-type, oxacillinase-48-type; KPC, *Klebsiella pneumoniae* carbapenemase.

Phenotypical enzyme production testing revealed an increased number of ESBL–CTX-M complex (*n* = 309, 90.88%) and carbapenemase (*n* = 306, 90.00%) producers. More than half (*n* = 171, 55.88%) of carbapenemase producers exhibited double enzyme activity on phenotypical assay, with NDM+OXA-48-type being the most common association. No VIM or IMP carbapenemases were detected in the isolates. A complete visual representation of carbapenemase distribution is found in Figure 1.

### 3.4 General antimicrobial resistance rates

Upon general antimicrobial susceptibility testing, the tested strains exhibited increased rates of resistance to all categories of antimicrobials, the highest being in penicillins, fluoroquinolones and 3rd generation cephalosporins (> 93%). Nonetheless, last-resort antimicrobials presented limited in-vitro activity against *Klebsiella pneumoniae*, with rates of resistance of over 70% in CZA, CZT, IMR and COL and 32.05% against cefiderocol. A more comprehensive dataset can be found in Table 2.

**TABLE 2:** Antimicrobial resistance rates toward different agents included in the usual Gram-negative AST panel as per laboratory protocol.

Antimicrobial class	Compound	Number of resistant isolates	Percentage of resistant isolates
Penicillins	Ampicillin-sulbactam (SAM)	334	98.23%
	Amoxicillin-clavulanic acid (AMC)	332	97.64%
	Piperacillin-tazobactam (TZP)	327	96.17%
3rd generation cephalosporins	Ceftazidime (CAZ)	318	93.52%
	Ceftriaxone (CRO)	329	96.76%
	Cefotaxime (CTX)	332	97.64%
Aminoglycosides	Amikacin (AMK)	267	78.52%
	Gentamycin (GEN)	276	81.17%
	Tobramycin (TOB)	296	87.05%
Fluoroquinolones	Ciprofloxacin (CIP)	330	97.05%
	Levofloxacin (LVX)	327	96.17%
Monobactams	Aztreonam (ATM)	319	93.82%
Novel beta-lactam/beta-lactamase inhibitors	Ceftazidime-avibactam (CZA)	257	75.58%
	Ceftolozane-tazobactam (CZT)	320	94.11%
Carbapenems/beta-lactamase inhibitors	Imipenem-relebactam (IMR)	254	74.70%
Polymyxins	Colistin (COL)	239	70.29%
Siderophore-cephalosporins	Cefiderocol (FDC)	109	32.05%

Further classification revealed that 25.00% (*n* = 85) of isolates were multidrug-resistant (MDR), 56.47% (*n* = 192) extensively drug-resistant (XDR) and 17.06% (*n* = 58) were pandrug-resistant (PDR). In addition, over half of XDR isolates demonstrated susceptibility to only one antimicrobial agent (54.17%, *n* = 104)—colistin or cefiderocol. Highest percentage of PDR and XDR strains were recorded in double NDM+OXA-48-type producers, as shown in Table 3.

**TABLE 3:** MDR/XDR/PDR classification per carbapenemase type.

Carbapenemase	MDR	XDR	Out of which, XDR with susceptibility to only one drug	PDR
NDM (*n* = 74)	12.16% (*n* = 9)	68.92% (*n* = 51)	35.29% (*n* = 18)	18.92% (*n* = 14)
KPC (*n* = 17)	64.71% (*n* = 11)	29.41% (*n* = 5)	0.00% (*n* = 0)	5.88% (*n* = 1)
OXA-48-type (*n* = 44)	75.00% (*n* = 33)	22.73% (*n* = 10)	30.00% (*n* = 3)	2.27% (*n* = 1)
NDM+OXA-48-type (*n* = 164)	3.05% (*n* = 5)	72.56% (*n* = 119)	68.91% (*n* = 82)	24.39% (*n* = 40)

### 3.5 Correlations between carbapenemase type and resistance rates in last-resort antimicrobials

Upon further investigation, we aimed to characterize the variation of resistance rates toward last-resort antimicrobials (FDC, CZA, CZT, IMR and COL) when different types of carbapenemases are concerned. Thus, we have calculated pooled resistance rates (PRR) for the four most common carbapenemases—NDM, OXA-48-type, KPC and NDM+OXA-48-type producing isolates. The highest pooled resistance rates occurred in NDM producers (PRR = 82.70%), followed by double NDM+OXA-48-type producers (PRR = 81.21%). KPC PRR was 49.41%, while OXA-48-type producing isolates presented a 47.27% pooled resistance rate.

NDM isolates exhibited higher resistance rates, when compared to the resistance rates of all included isolates, to all but one (colistin) last-resort agents (47.29% versus 32.05% for cefiderocol, *p* = 0.01; 100% versus 75.58% for ceftazidime-avibactam, *p* < 0.01; 100% versus 94.11% for ceftolozane-tazobactam, *p* < 0.01; 98.64% versus 74.70% for imipenem-relebactam, *p* < 0.01). Similarly, isolates harboring both NDM and OXA-48-type showed higher-than-general resistance rates to all last-resort agents (97.56% versus 75.58% for ceftazidime-avibactam, *p* < 0.01; 98.78% versus 94.11% for ceftolozane-tazobactam, *p* = 0.03; 98.17% versus 93.82% for aztreonam, *p* = 0.054; 99.39% versus 74.70% for imipenem-relebactam, *p* < 0.01; and 80.48% versus 70.29% for colistin, *p* = 0.02), except cefiderocol (29.87% versus 32.05%, *p* = 0.69).

**FIGURE 2:**
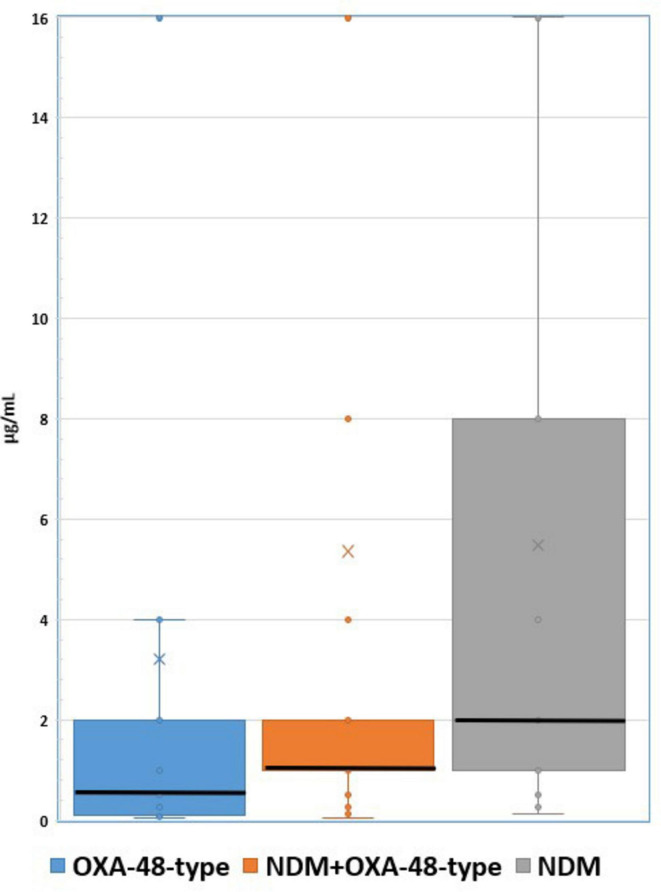
Cefiderocol MIC distribution per each subgroup.

Regarding cefiderocol resistance, our results showed that KPC positive isolates had the highest resistance rates (64.70%), followed by NDM (47.29%), NDM+OXA-48-type (29.87%) and OXA-48-type (11.36%). Median minimum inhibitory concentrations (MICs) with a 95% confidence interval (CI) for FDC were calculated for NDM, NDM+OXA-48-type and OXA-48-type isolates, the results being as follows: median MIC of 2 μg/mL in NDM isolates, 1 μg/mL in NDM+OXA-48-type isolates and 0.5 μg/mL in OXA-48-type strains (Figure 2).

**FIGURE 3:**
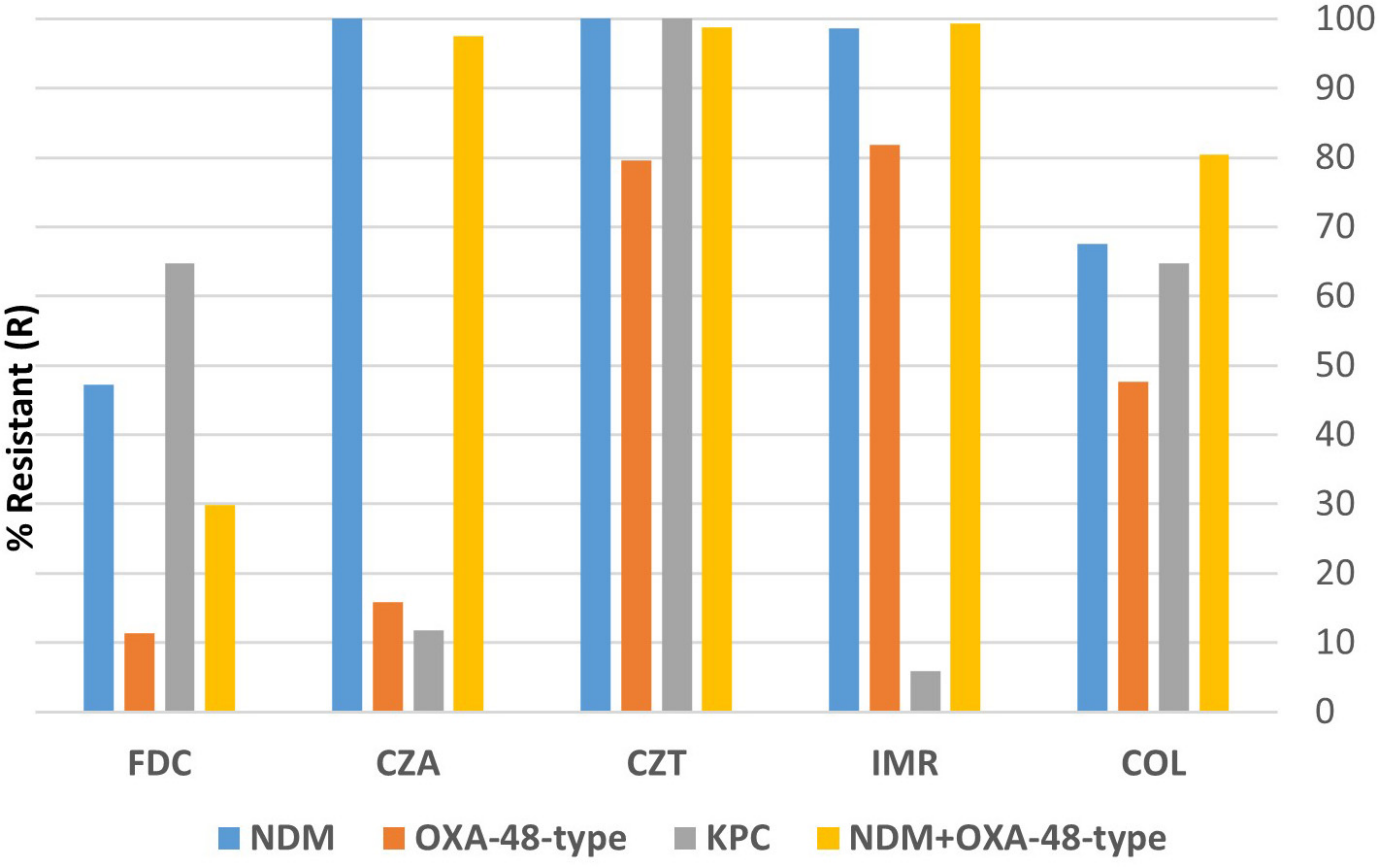
Resistance rates to last-resort antimicrobials amongst different carbapenemases producers. Values shown in % resistant (R). FDC, cefiderocol; CZA, ceftazidime-avibactam; CZT, ceftolozane-tazobactam; IMR, imipenem-relebactam; COL, colistin. Complete resistance values per carbapenemase are found in the Supplementary Table 1.

A visual representation of resistance rates per antimicrobial considering each mentioned carbapenemase may be found in Figure 3, with the numerical values available in the Supplementary Table 1.

## 4 Discussion

While *Klebsiella pneumoniae* may exhibit a high variety of resistance mechanisms against carbapenems, the production of carbapenemases still stands as the leading cause, with 90.00% of the strains included in our study showcasing phenotypic ability of enzyme production. In the absence of molecular characterisation of isolates and taking into account that phenotypical assays do not have 100% sensitivity for each carbapenemase, it is fair to assume that carbapenem resistance in the remaining 34 strains occurs through either carbapenemase-encoding genes or other mechanisms, such as decreased antimicrobial permeability or intensified efflux pump activity (Aslam et al., 2020; Aurilio et al., 2022; Elshamy and Aboshanab, 2020). While we have expected resistant rates to more common antimicrobials to be increased, special attention goes into the results concerning last-resort antimicrobials.

These results further emphasize the ongoing impact of NDM-producers, as this Amber Class B enzyme is able to inactivate ceftazidime-avibactam, ceftolozane-tazobactam and imipenem-relebactam with utmost efficiency (Van Duin and Bonomo, 2016). Moreover, the fact that double carbapenemase producers are now starting to dominate the landscape concerning CRKP, imposes a significant challenge on healthcare providers, who are already struggling with limited treatment options in increasingly severe cases (Lazar et al., 2024). Studies concerning isolates from Eastern Europe provide supporting evidence that NDM+OXA-48-type producing CRKP are increasing in terms of prevalence throughout many states, such as Romania (Lazar et al., 2024; Melinte et al., 2025) or Ukraine (Zwittink et al., 2022; Schultze et al., 2023; Biedrzycka et al., 2025), a stark contrast from Western European isolates, which show limited carbapenem resistance and production of enzymes, predominantly NDM (Zwittink et al., 2022). Meanwhile, other countries affected by CRKP, such as Greece, report KPC as the most commonly encountered carbapenemase produced (Tryfinopoulou et al., 2023; Tsolakidou and Chatzidimitriou, 2025).

Cefiderocol, previously considered a promising treatment alternative, has been introduced in our hospital in March 2024, its use being limited to selected cases. Even so, the overall resistance rate toward cefiderocol in our isolates stands at over 30%, a worrisome figure, amplified by the finding that over 60% of KPC-producing isolates were deemed to be non-susceptible. Further studies are needed for in-depth characterisation of the resistance mechanisms to cefiderocol in KPC-producing strains.

Another observation of this study features the role of carbapenemase interaction into cefiderocol resistance, as explained both numerically and by MIC distribution amongst NDM, OXA-48-type and NDM+OXA-48-type isolates, suggesting that NDM has an increasing effect on cefiderocol MIC in double carbapenemase producers, while the opposite may be applied for the acquisition of OXA-48- type. Available sources indicate that the harboring of *bla*_NDM_ impacts cefiderocol susceptibility in a larger manner than *bla*_OXA–48_, which has limited hydrolysis capacity, thus partially explaining the discrepancies (Yang et al., 2024; Fröhlich et al., 2022). Moreover, NDM-producing strains have been more often associated with siderophore receptor and porin alterations on a molecular level, implying that carbapenemase production is only an “iceberg tip” in cefiderocol resistance, with an especially mentioned mutation concerning the *CirA* catecholate siderophore receptor enabling NDM hydrolysis to exponentially increase cefiderocol MIC (Tristancho-Baró et al., 2025; Lan et al., 2022; McElheny et al., 2021). However, there are limited sources available on the molecular interactions between *bla*_NDM_ and *bla*_OXA–48_, thus further research is needed in order to fully understand how each may impact the other in the virulence, pathogenicity and resistance profiling of the “superbugs” double carbapenemase producers constitute.

Nonetheless, we reiterate the role of antimicrobial stewardship in daily practice, not only against CRKP, but as a general principle, ensuring limited usage of last-resort antimicrobials, following the clinical flow of antibiotic therapy de-escalation.

### 4.1 Limitations

While our study provides clear observations, there are several limitations that need to be taken into account. Firstly, as this study was conducted in an infectious-diseases oriented center, antimicrobial resistance rates may be higher than in other clinical settings, possibly leading to overestimation and lack of scalability for a clear epidemiological point of view in terms of the burden antimicrobial resistance imposes over the healthcare system. Thus, generalizability of this study is limited. However, the present data are an everyday reality for our peers in the clinic. Another significant limitation is construed by the lack of molecular characterisation of assessed strains, which would have been an insightful tool into carbapenemase interactions and production.

## 5 Conclusion

Hereby, we provided an updated overview into carbapenem resistance—distribution, antimicrobial susceptibility rates and correlations amongst *Klebsiella pneumoniae* strains isolated from our hospital in Romania over the course of two years. In a landscape dominated by double carbapenemase producers in 55.88% of cases, exhibiting increased pooled resistance rates toward last-resort antimicrobials, limited therapeutic options emerge, with more than 70% resistance rate to ceftazidime/avibactam, ceftolozane/tazobactam, imipenem/relebactam and colisting, paired with a 32.05% resistance rate to cefiderocol. Metallo-β-lactamase exhibiting isolated tend to enable multiple resistance mechanisms, correlating with the highest pooled resistance rates (82.70%), as well as presumed influential interplay in double NDM+OXA-48-type producers. Albeit several limitations of the study need to be taken into account, the results further emphasize the issue CRKP represents for fellow clinicians dealing with difficult-to-treat infections, urging the need for novel antimicrobials development and more reliable implementation of antimicrobial stewardship measures.

## Data Availability

The raw data supporting the conclusions of this article will be made available by the authors, without undue reservation.
