# Supplementary material for: Insights into Klebsiella pneumoniae carbapenem resistance—a two-year retrospective study from a Romanian tertiary care hospital
Source: Front Microbiol. 2026 Jan 8;16:1728843. doi: 10.3389/fmicb.2025.1728843 (PMC12823975; doi:10.3389/fmicb.2025.1728843)
Supplement: Supplementary file 1 [file Table_1.docx]

**Supplementary material**

Supplementary Table 1. Numerical values of antimicrobial resistance rates towards last-resort agents in each carbapenemase category.

| Carbapenemase/Antimicrobial | FDC resistance | CZA resistance | CZT resistance | IMR resistance | COL resistance |
| --- | --- | --- | --- | --- | --- |
| NDM (N=74) | 47.29% (n=35) | 100.00% (n=74) | 100.00% (n=74) | 98.64% (n=73) | 67.56% (n=50) |
| OXA-48-type (N=44) | 11.36% (n=5) | 15.90% (n=7) | 79.54% (n=35) | 81.81% (n=36) | 47.72% (n=21) |
| KPC (N=17) | 64.70% (n=11) | 11.76% (n=2) | 100% (n=17) | 5.88% (=1) | 64.70% (n=11) |
| NDM + OXA-48-type (N=164) | 29.87% (n=49) | 97.56% (n=160) | 98.78% (n=162) | 99.39% (n=163) | 80.48% (n=132) |
